# CDK-Dependent Hsp70 Phosphorylation Controls G1 Cyclin Abundance and Cell-Cycle Progression

**DOI:** 10.1016/j.cell.2012.10.051

**Published:** 2012-12-07

**Authors:** Andrew W. Truman, Kolbrun Kristjansdottir, Donald Wolfgeher, Naushaba Hasin, Sigrun Polier, Hong Zhang, Sarah Perrett, Chrisostomos Prodromou, Gary W. Jones, Stephen J. Kron

**Affiliations:** 1 Ludwig Center for Metastasis Research, The University of Chicago, Chicago, IL 60637, USA; 2 Department of Molecular Genetics and Cell Biology, The University of Chicago, Chicago, IL 60637, USA; 3 Department of Biology, National University of Ireland Maynooth, Maynooth, County Kildare, Ireland; 4 Genome Damage and Stability Centre, Science Park Road, University of Sussex, Brighton BN1 9RQ, England; 5 National Laboratory of Biomacromolecules, Institute of Biophysics, Chinese Academy of Sciences, Beijing 100101, China

## Abstract

In budding yeast, the essential functions of Hsp70 chaperones Ssa1–4 are regulated through expression level, isoform specificity, and cochaperone activity. Suggesting a novel regulatory paradigm, we find that phosphorylation of Ssa1 T36 within a cyclin-dependent kinase (CDK) consensus site conserved among Hsp70 proteins alters cochaperone and client interactions. T36 phosphorylation triggers displacement of Ydj1, allowing Ssa1 to bind the G1 cyclin Cln3 and promote its degradation. The stress CDK Pho85 phosphorylates T36 upon nitrogen starvation or pheromone stimulation, destabilizing Cln3 to delay onset of S phase. In turn, the mitotic CDK Cdk1 phosphorylates T36 to block Cln3 accumulation in G2/M. Suggesting broad conservation from yeast to human, CDK-dependent phosphorylation of Hsc70 T38 similarly regulates Cyclin D1 binding and stability. These results establish an active role for Hsp70 chaperones as signal transducers mediating growth control of G1 cyclin abundance and activity.

## Graphical abstract

**Graphical Abstract:**

**Figure:**
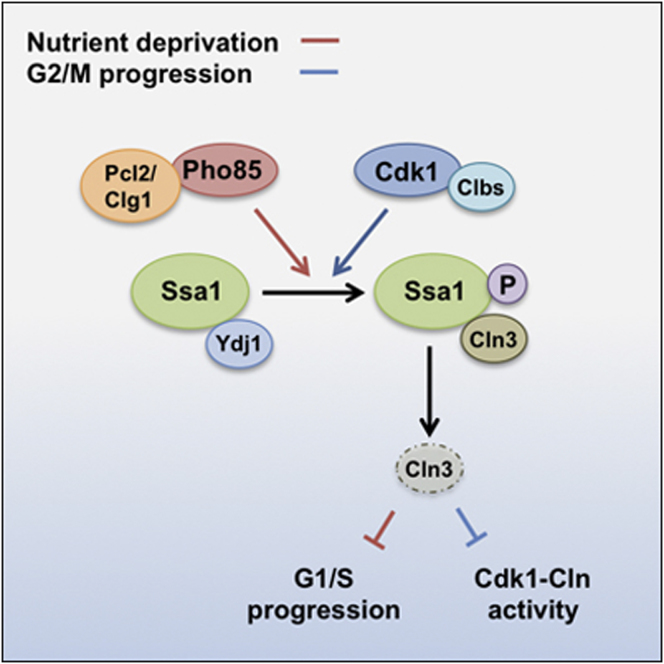

**Highlights:**

► First demonstration of regulation of Hsp70 function through CDK phosphorylation ► Cdk1 and Pho85 phosphorylate Ssa1 to regulate binding to Cln3 and Cln3 destruction ► Cln3 degradation requires *both* Cln3 phosphorylation and Cln3 binding to Ssa1 ► CDK-mediated phosphorylation of Hsc70 targets Cyclin D1 abundance and activity

## Teaser

Cyclin-dependent kinases directly phosphorylate yeast Hsp70 to enhance its interaction with Cyclin 3, triggering its destruction and the G1/S checkpoint, showing that chaperone proteins can act as signal transducers during cell-cycle progression.

## Introduction

The 70 kDa heat shock proteins (Hsp70s) are highly conserved and ubiquitous molecular chaperones essential for cell viability. They bind a range of client proteins, directing key events in their life cycle from folding to destruction (Craig and Marszalek, 2011; Mayer and Bukau, 2005; Meimaridou et al., 2009). Hsp70 proteins share an N-terminal ATPase domain, a substrate binding domain and a C-terminal regulatory domain that mediates cochaperone interaction (Mayer and Bukau, 2005). The budding yeast *Saccharomyces cerevisiae* genome encodes four functionally redundant cytosolic Hsp70s, Ssa1–4, which differ in expression pattern but are together essential for cell viability (Newcomb et al., 2003).

The yeast DnaJ-related cochaperones (J-proteins) Ydj1 and Sis1 regulate Hsp70 ATP hydrolysis and client interactions (Brehmer et al., 2001; Kampinga and Craig, 2010). Interestingly, despite partially overlapping functions, only Sis1 is essential (Caplan and Douglas, 1991; Jones et al., 2004).

Among many known functions of yeast Hsp70 and J-domain proteins, a complex role in cell proliferation has been described. One Ssa client is the cyclin Cln3, the yeast homolog of Cyclin D. Cln3 accumulation activates the cyclin-dependent kinase (CDK) Cdk1 to phosphorylate Whi5. This releases SBF (Swi4-Swi6) and MBF (Swi6-Mbp1) to promote Cln1 and Cln2 cyclin expression, G1 exit, and S phase onset (de Bruin et al., 2004; Ferrezuelo et al., 2010; Sherlock and Rosamond, 1993). Diverse stress signals, including mating pheromone stimulation and nitrogen starvation can delay G1/S progression by promoting phosphorylation of PEST sequences in Cln3 leading to proteasome-mediated Cln3 degradation (Benanti, 2012). Significantly, Ssa proteins and Ydj1 are required for normal Cln3 phosphorylation and destruction (Vergés et al., 2007; Yaglom et al., 1995; Yaglom et al., 1996). Unlike other Ssa clients, Cln3 encodes a J-like domain that competes with Ydj1 for binding. As a result, Ssa proteins can sequester accumulating Cln3 on the ER in early G1. Then, Ydj1 displaces Cln3 in late G1, allowing its transit to the nucleus to drive cell cycle progression (Vergés et al., 2007). The mechanism of this switch remains poorly understood.

A second yeast CDK, Pho85, has a special role in responding to stress signals by targeting multiple survival, morphogenesis, and proliferation pathways (Carroll and O’Shea, 2002; Huang et al., 2007; Yang et al., 2010). Pho85 cyclins (Pcls) direct the kinase to specific targets, including substrates shared with Cdk1 (Huang et al., 1998; Mazanka and Weiss, 2010). Like Cln3, Pcl9 induces Pho85 phosphorylation of Whi5 in early G1 (Huang et al., 2009; Kung et al., 2005) to promote Cln1/2 and Pcl1 expression. Then, like Cln1/2, Pcl1 targets Pho85 to Sic1 in late G1 (Nishizawa et al., 1998; Wysocki et al., 2006) to release Cdk1-Clb activity in S phase.

Despite proteomic evidence of extensive Ssa phosphorylation and the recent finding that mutation of putative phosphorylation sites affects essential functions (Beltrao et al., 2012), a regulatory role remains to be established. However, cell-cycle-dependent phosphorylation of yeast Hsp90 alters chaperone-client interactions, suggesting functional significance (Mollapour et al., 2010). Here, we show that Ssa1 can be phosphorylated by Cdk1 or Pho85 on T36, a CDK consensus site conserved across the Hsp70 family. T36 phosphorylation displaces Ydj1 to allow binding of Cln3, leading to its degradation. By slowing accumulation of Cdk1-Cln3, Ssa1 T36 phosphorylation prevents inactivation of Whi5 and delays onset of Cln1/2 expression. Establishing a key role for Ssa proteins in cell-cycle arrest in response to cell stress, upon pheromone stimulation or nitrogen starvation, Pho85 bound to Clg1 and/or Pcl2 targets T36 to block G1/S progression. Notably, CDK-dependent T38 phosphorylation on mammalian Hsc70 similarly regulates Cyclin D1 binding and activity. Our results support a view of Hsp70 chaperones as active regulators of cell division, integrating environmental cues along with cellular events to help make the critical decision in G1 to proliferate or arrest.

## Results

### Cell Proliferation Defects in Ssa1 T36 Mutants

**Figure 1:**
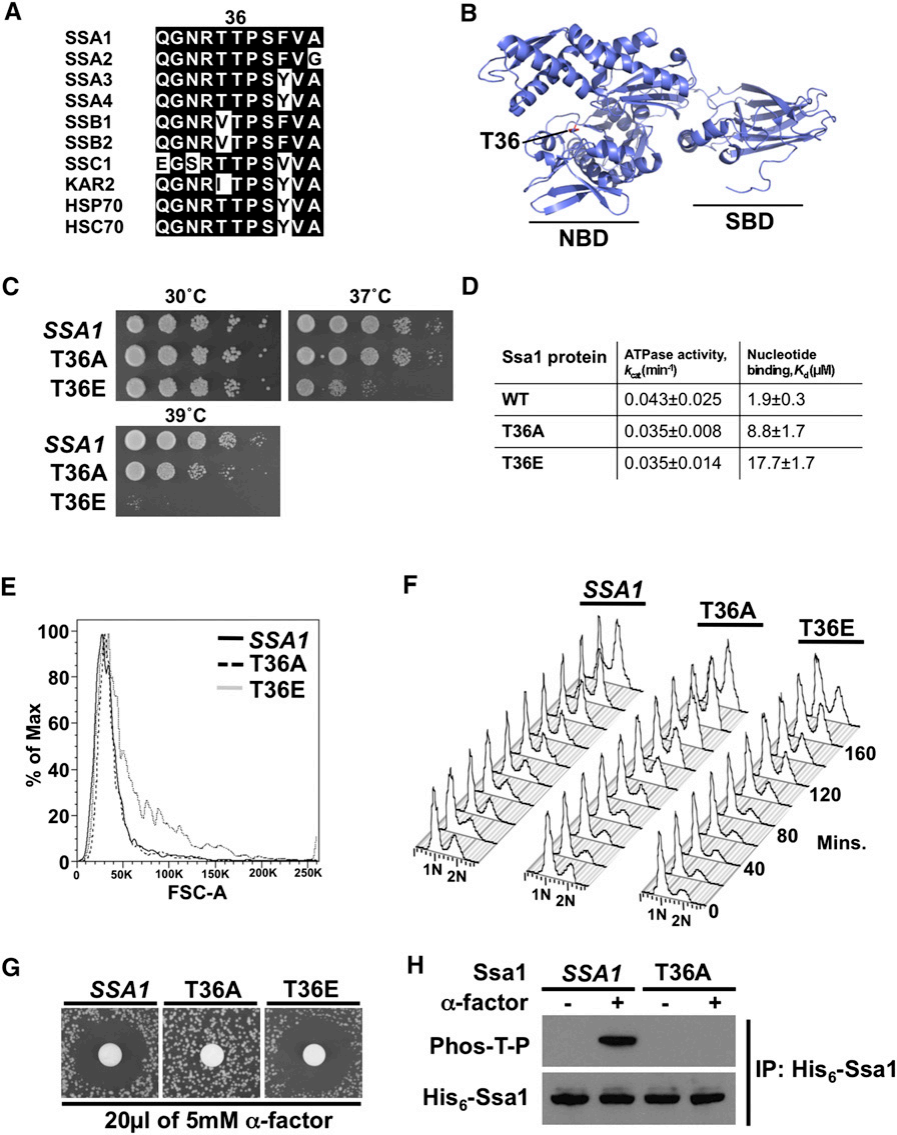
Mutation of a Conserved Threonine in the N Terminal of Ssa1 Affects Chaperone Function (A) Alignment of Hsp70 proteins from yeast and human cells. A cyclin-dependent kinase consensus (S/TP) site at T36 in Ssa1 is conserved across the yeast Hsp70s and among mammalian Hsp70 isoforms (including Hsp70 and Hsc70 as shown here). (B) T36 resides adjacent to the ATP-binding pocket on Hsc70. T36 is marked in red on the Hsc70 structure (PDB: 1YUW). NBD is nucleotide-binding domain, SBD is substrate binding domain. (C) Temperature sensitivity of the Ssa1 T36 phosphomutants. Cells expressing wild-type Ssa1 (*SSA1*) or Ssa1 phosphosite mutants T36A or T36E as their sole Ssa protein were spotted in serial 10-fold dilutions onto YPD media and were incubated for 48 hr at 30°, 37° or 39°C. (D) Mutation of T36 affects nucleotide binding. The intrinsic ATPase activity of WT, T36A and T36E Ssa1 was assayed and the *k*_cat_ values (min^-1^) are shown as mean ± SD (n = 3). Nucleotide binding of WT, T36A and T36E was calculated by isothermal calorimetry. The *K*_d_ (μM) for the Ssa1 proteins are shown as mean ± SD (n = 3). (E) Ssa1 T36E mutation confers a large G1 cell phenotype. Cells grown at 30**°** C to mid-log phase were analyzed by flow cytometry. Cells in G1 stage of cell cycle were gated based on 1N DNA content, and the size distribution based on Forward Scatter (FSC) of each strain is plotted. (F) T36E cells display delayed G1/S progression. Cells were synchronized in G1 by nitrogen starvation then released into rich media and incubated at 30**°** C. At 20 min intervals, cells were fixed and assessed for DNA content by flow cytometry. (G) Halo assay of response of *SSA1*, T36A and T36E to αF pheromone reveals an arrest defect in T36A, expressing the nonphosphorylatable Ssa1 mutant. (H) Ssa1 T36 is phosphorylated in response to αF. His_6_-Ssa1 was purified from untreated or αF-treated *SSA1* or T36A cells. Ssa1 phosphorylation was assessed with CDK phosphosite-specific anti-Phos-T-P antibody. See also Figure S1.

Global phosphoproteomic analysis has identified 18 phosphorylation sites in Ssa proteins (Albuquerque et al., 2008) but biological significance has yet to be established. Of these phosphosites, only T36 lies within a consensus S/T-P CDK phosphorylation motif. T36 is highly conserved among Hsp70 family proteins (Figure 1A) and is situated in the N-terminal domain, proximal to the ATP binding and cochaperone binding region (Figure 1B), suggesting a potential regulatory role.

**Figure S1:**
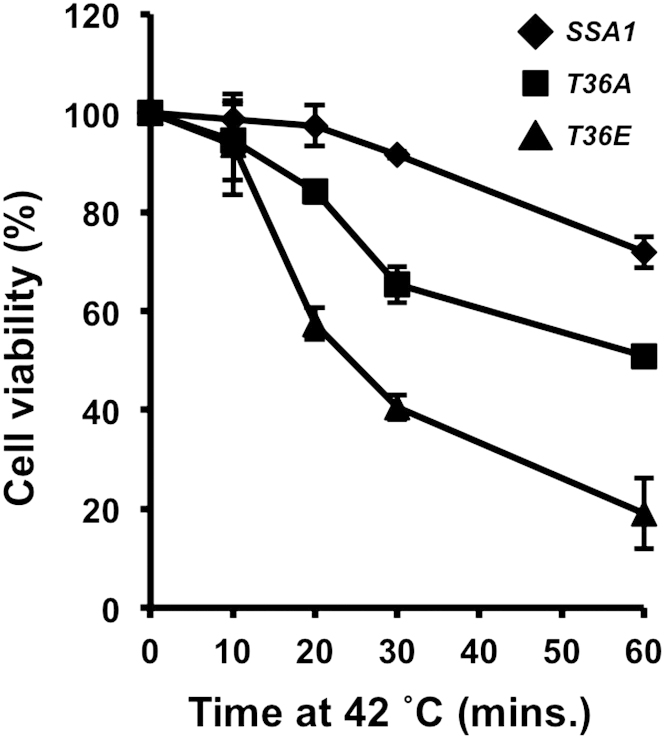
T36A and T36E Cells Lose Clonogenicity at 42°C Compared to *SSA1*, Related to Figure 1 *SSA1, T36A* and *T36E* cells grown at 30°C in YPD media to an OD_600_ of 0.5. Aliquots of cells were incubated at 42°C for the indicated times, were plated onto YPD plates and incubated for 2 days at 30°C. Colonies were counted and percentage cell viability was determined. Data shown are the mean and SD (n = 3).

To examine functions of T36 phosphorylation, we used a yeast strain in which all four *SSA* genes have been deleted and functionally complemented by *SSA1* expressed from a *URA3-*marked centromeric plasmid (Jaiswal et al., 2011). The strain was transformed with a *LEU2*-marked plasmid bearing either wild-type *SSA1*, the nonphosphorylatable mutant *ssa1-T36A*, or the phosphomimetic mutant *ssa1-T36E*. The *URA3* plasmid was evicted on 5-fluoro-orotic acid (5-FOA) media to yield strains expressing wild-type Ssa1, Ssa1-T36A (mimic for nonphosphorylated form), or Ssa1-T36E (mimic for phosphorylated form) as the sole Ssa protein in the cell, hereafter referred to as *SSA1*, T36A, and T36E cells. The *SSA1*, T36A, and T36E cells were similarly viable at 30°C, but the T36E mutation conferred impaired growth at 37°C and inviability at 39°C (Figure 1C). Both T36A and T36E cells demonstrated increased sensitivity to incubation at 42°C (Figure S1 available online).

We analyzed the effect of T36 mutation on the in vitro properties of Ssa1. Whereas intrinsic ATPase activity of WT, T36A, and T36E proteins was not significantly altered, nucleotide binding was decreased in both mutants, suggesting an important regulatory role for this site in Ssa1 function (Figure 1D).

To further dissect effects on cell proliferation, we examined the growth of *SSA1*, T36A, and T36E cells as they progressed through the cell cycle. In asynchronous growth, T36E cells attained a larger cell size before exiting G1 compared to *SSA1* or T36A cells (Figure 1E), suggesting a role for Ssa phosphorylation in delaying S phase onset. Indeed, flow cytometry of cells synchronized in G1 by nitrogen starvation and released into fresh media revealed that while most *SSA1* and T36A cells progressed toward 2N DNA content by 80 to 100 min after release, many T36E cells remained in G1 past 120 min (Figure 1F). In turn, when *SSA1*, T36A, and T36E cells were exposed to the peptide mating pheromone α-Factor (αF), T36A cells failed to arrest, unlike *SSA1* or T36E (Figure 1G). To examine Ssa1 T36 phosphorylation in response to αF, we generated strains expressing hexahistidine-tagged WT or T36A as the sole Ssa protein in the cell. The His_6_-*SSA1* and -T36A cells were treated with αF, and His_6_-Ssa1 protein was purified. Immunoblotting with anti-phospho-Thr-Pro antibody (anti-Phos-T-P, specific to consensus CDK phosphosites) demonstrated αF-dependent Ssa1 phosphorylation, absent from T36A (Figure 1H). These data suggest that T36 is phosphorylated in response to pheromone stimulation and may have a specific role in G1/S cell cycle control.

### Proteomic Analysis of Phosphoregulation of the Ssa1 Interactome

**Figure 2:**
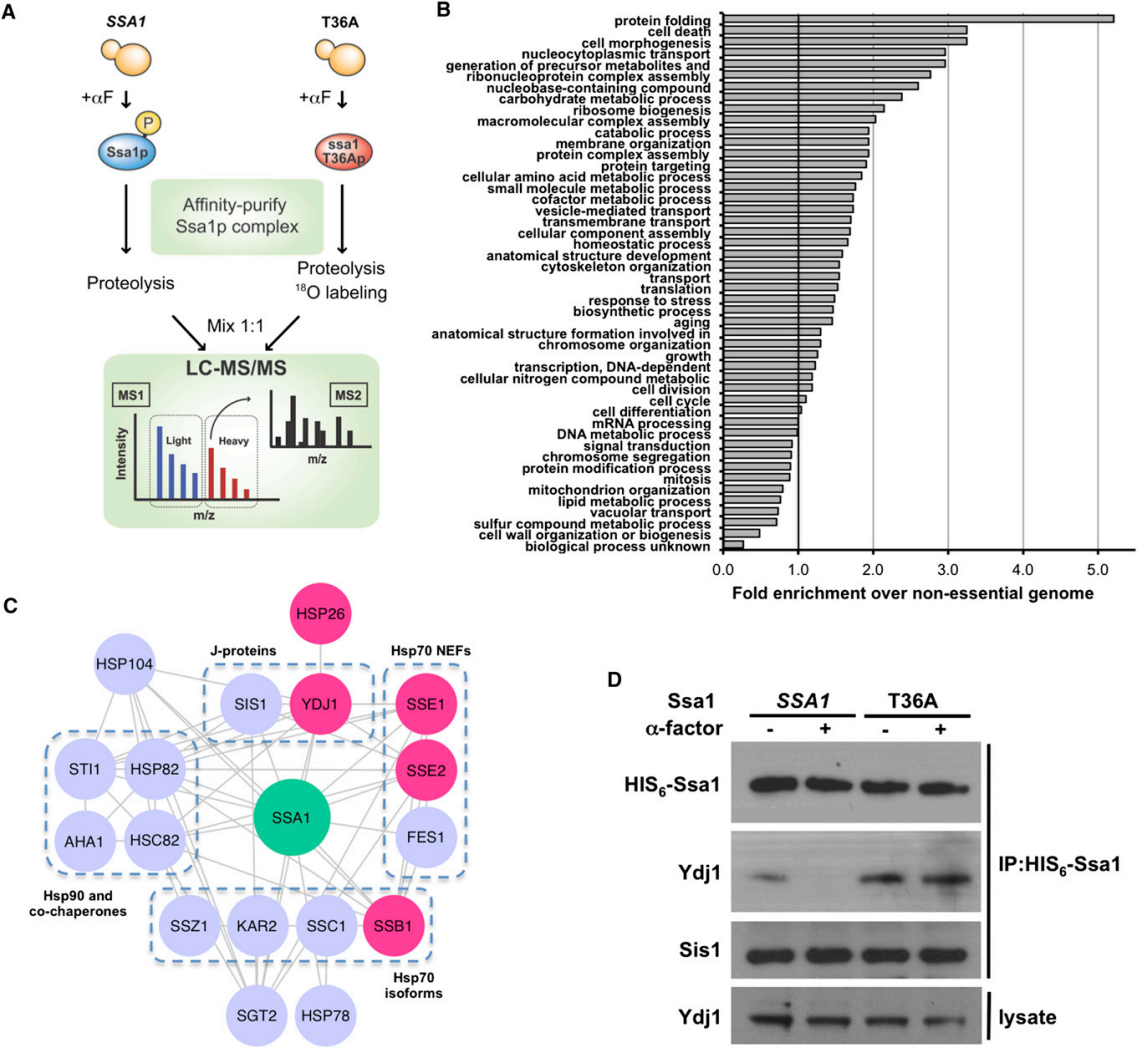
Phosphorylation Alters the Ssa1 Interactome (A) Scheme for proteomic analysis of Ssa1 interactome. Cells expressing wild-type (*SSA1*) or T36A mutant His_6_-Ssa1 were exposed to αF. Ssa1 complexes were affinity purified and digested. Peptides from Ssa1-T36A interactors were isotopically labeled with ^18^O, mixed 1:1 with wild-type Ssa1 interactor peptides, and analyzed by quantitative LC-MS/MS. (B) Functional classification of the Ssa1 interactome. Ssa1 interactors were categorized by cellular function using GO Slim analysis, and plotted by relative enrichment compared to occurrence in the nonessential genome. (C) Chaperones and cochaperones in the Ssa1 interactome. The 18 chaperone/cochaperone proteins identified were analyzed for interactions using STRING and visualized by Cytoscape, grouped by homology and function. Pink nodes represent proteins that decreased interaction with Ssa1 upon T36 phosphorylation. (D) Differential effect of T36 phosphorylation on Sis1 and Ydj1 binding. His_6_-Ssa1 was pulled down from WT and T36A cells and probed for Ydj1 or Sis1 coprecipitation. See also Table S1 and Figure S2.

**Figure S2:**
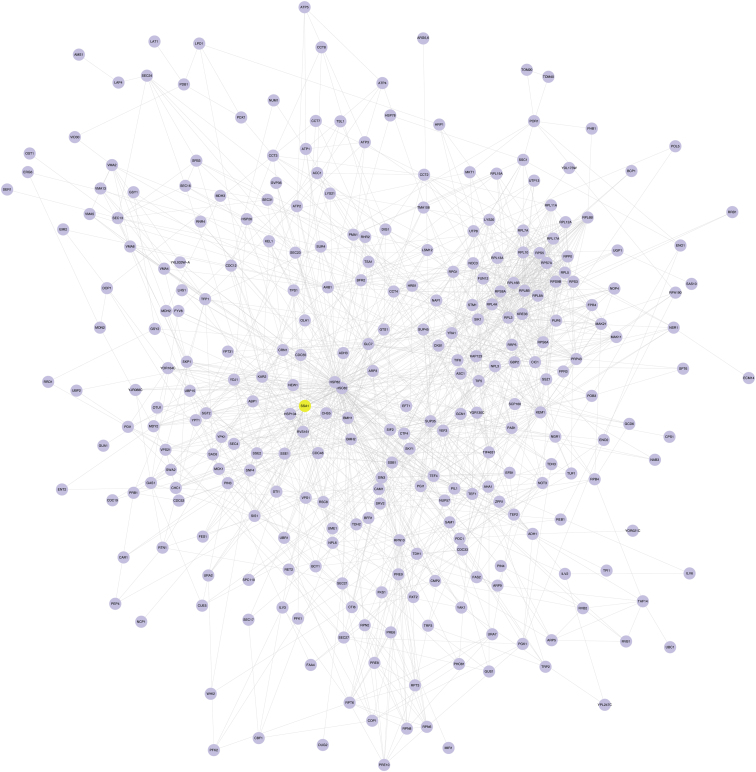
Interactome Map for Ssa1, Related to Figure 2 Known protein-protein interactions were calculated by processing the data obtained from Ssa1 Mass spectrometry studies through STRING (*string-db.org)* and are displayed using Cytoscape. Ssa1 is shown in yellow.

Toward identifying Ssa1 partners regulated by T36 phosphorylation, we treated the His_6_-*SSA1* and T36A cells with αF, purified the His_6_-Ssa1 interactomes and compared them by isotope-coded tandem mass spectrometry (LC-MS/MS, Figure 2A). Gene Ontology (GO) analysis of 317 candidate Ssa partners, identified based on high confidence peptide matches, revealed significant enrichment of multiple cellular functions (Figure 2B; Table S1; Figure S2). The GO term “protein folding*”* was most enriched, reflecting the wide range of chaperones and cochaperones identified (Figure 2C). Other enriched categories include “cell death” and “morphogenesis,” “protein transport,” “metabolism,” and “ribosome biogenesis.” Proteins that displayed ^18^O:^16^O peptide ratios >> 1 were inferred to be candidates for interaction partners that dissociate from Ssa1 upon T36 phosphorylation (Figure 2C, pink). Among chaperones/cochaperones known to directly bind Ssa1, those displaying the highest ratios were the ribosomal Hsp70 Ssb1, the Hsp110 homologs Sse1 and 2 (Hsp70 nucleotide exchange factors), the J-protein Ydj1, and Hsp26. Of these, Ydj1 has previously been ascribed a key role in G1/S cell cycle regulation (Yaglom et al., 1995; Vergés et al., 2007).

To directly test Ydj1 dissociation upon T36 phosphorylation, we isolated His_6_-WT and T36A proteins from cells treated with αF. Western blotting indicated that T36 phosphorylation antagonizes Ydj1 binding to Ssa1 without affecting the essential J-protein Sis1 in *SSA1* cells, whereas Ydj1 and Sis1 binding are similarly insensitive to pheromone stimulation in T36A cells (Figure 2D).

### Mutation of Ssa1 T36 Affects Cln3 Accumulation and Functions

**Figure 3:**
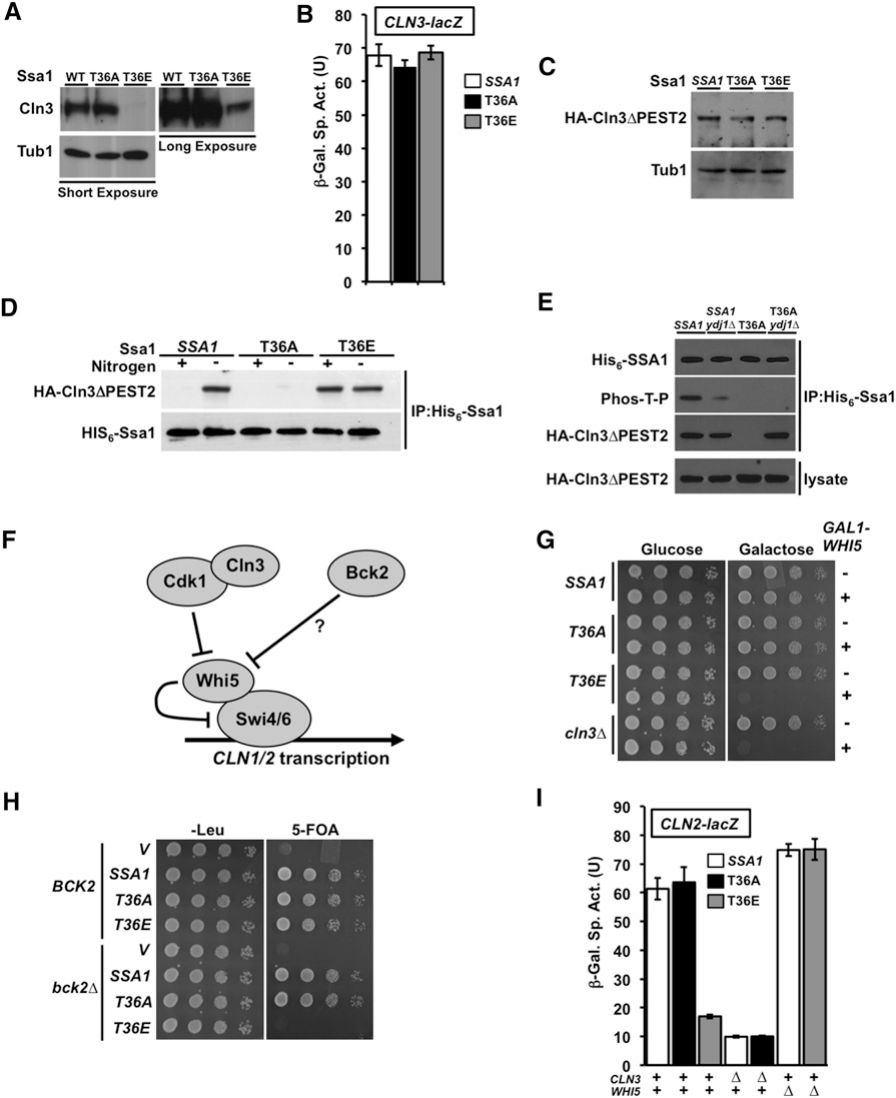
Interaction of T36 Mutants with Cln3 and the G1/S Transcription Pathway (A) Western blotting of cell extracts reveals decreased Cln3 protein levels in T36E cells. A long exposure reveals residual expression in T36E. (B) Mutation of T36 does not alter *CLN3* transcription. *SSA1*, T36A and T36E cells transformed with a *CLN3-lacZ* reporter were assessed for *CLN3* transcription. Each value represents mean ± SD (n = 3). (C) Cln3 lacking PEST domains displays similar abundance in *SSA1*, T36A and T36E cells, indicating the effect of T36E is mediated by ubiquitin-proteasome degradation. (D) Ssa1 phosphorylation regulates Cln3 binding. HIS_6_-Ssa1-WT, T36A and T36E were purified from cells expressing HA-Cln3ΔPEST2. Immunoblotting reveals Cln3 binding induced by nitrogen starvation in *SSA1* is absent in T36A and constitutive in T36E. (E) Dissociation of Ydj1 from Ssa1 upon T36 phosphorylation allows Cln3 to bind Ssa1. Ydj1 wild-type or deletion mutant cells expressing either His_6_-Ssa1 WT or T36A, along with stabilized, PEST mutant Cln3 were treated with 15 μg/ml nocodazole for 3 hr. Ssa1 was pulled down to determine T36 phosphorylation by anti-Phos-T-P blotting and assess Ssa1-Cln3 interaction by coprecipitation of HA-Cln3ΔPEST2. (F) Schematic of regulation of G1/S transcription by Cdk1-Cln3 and Bck2. (G) Overexpression of Whi5 is lethal in T36E cells. *SSA1*, T36A or T36E cells transformed with either *GAL1*-WHI5-FLAG or empty vector pRS316 were spotted in serial 10-fold dilutions on either glucose or galactose medium and incubated for 48 hr at 30°C. (H) Bck2 is essential for cell viability in T36E cells. Both *ssa1-4*Δ and *ssa1-4*Δ *bck2*Δ cells were transformed with *LEU2*-marked plasmids carrying *SSA1*, *ssa1*-*T36E* or *ssa1*-*T36E* or empty vector control (V) and then spotted in serial 10-fold dilutions on either YPD or 5-FOA media to evict a *URA3*-marked wild-type *SSA1* plasmid, then incubated for 48 hr at 30°C. (I) T36E cells are defective in *CLN2* expression. *SSA1*, T36A and T36E cells, bearing deletions of *CLN3* or *WHI5* as indicated, were transformed with a *CLN2-lacZ* plasmid and *CLN2* expression measured by β-galactosidase assay. Each value represents the mean ± SD (n = 3). See also Figure S3.

The J-protein Ydj1 and the G1 cyclin Cln3 display reciprocal patterns of binding to Ssa proteins (Vergés et al., 2007), suggesting that Ssa1 T36 phosphorylation might affect Cln3 activity or expression. Western analysis revealed a striking decrease in Cln3 protein abundance in T36E cells, although residual Cln3 was detected in longer exposures (Figure 3A). *CLN3* transcription was not affected by T36 mutation (Figure 3B), indicating a posttranslational mechanism. Consistent with regulation via ubiquitin-proteasome-mediated degradation, a Cln3 mutant lacking the carboxyl-terminal PEST domains was similarly stable in *SSA1*, T36A, and T36E (Figure 3C).

Vergés et al. (2007) describe a mechanism by which binding of Ydj1 to Ssa1 promotes release of Cln3. We considered whether this process might be regulated by T36 phosphorylation. Pull-down of His_6_-Ssa1 revealed binding of stabilized Cln3 can be induced by nitrogen starvation in *SSA1*, but is lost from T36A, and becomes constitutive in T36E (Figure 3D). Suggesting that T36 phosphorylation disrupts the Ydj1-Ssa1 interaction to allow Cln3 access, loss of Ydj1 (*ydj1*Δ) restored Cln3 binding to Ssa1 in nitrogen-starved T36A cells (Figure 3E). Interestingly, in *SSA1* cells lacking Ydj1, T36 phosphorylation upon nitrogen starvation was attenuated.

As a functional test of Cln3 activity, we expressed Whi5, a Cdk1-Cln3 target and repressor of G1/S transcription (Figure 3F), in *SSA1*, T36A, T36E, and *cln3*Δ cells under control of a strong, galactose-inducible promoter. Overexpression of Whi5 in cells lacking Cln3 function results in loss of viability (Costanzo et al., 2004). Like the *cln3*Δ control, the T36E mutant was unable to grow on galactose media, suggesting a critical deficit of Cdk1-Cln3 activity (Figure 3G). Similarly, we examined genetic interactions of the Ssa1 T36 mutants with Bck2, a positive regulator of G1/S transcription (Di Como et al., 1995; Ferrezuelo et al., 2009; Wijnen and Futcher, 1999). Cln3 and Bck2 serve partly redundant, essential functions. As such, *bck2*Δ *cln3*Δ double mutant cells are unable to transition to S phase, arresting in late G1 (Di Como et al., 1995; Ferrezuelo et al., 2009; Wijnen and Futcher, 1999). Whereas *SSA1* and T36A cells lacking Bck2 remained viable, T36E was synthetically lethal with the *bck2*Δ mutation (Figure 3H). Consistent with these results, *CLN2* expression was decreased significantly in T36E, nearly to the level of a *cln3*Δ mutant but was restored in the *whi5*Δ background (Figure 3I).

### Ssa1 T36 Is Phosphorylated by Cyclin-Dependent Kinases Cdk1 and Pho85

**Figure 4:**
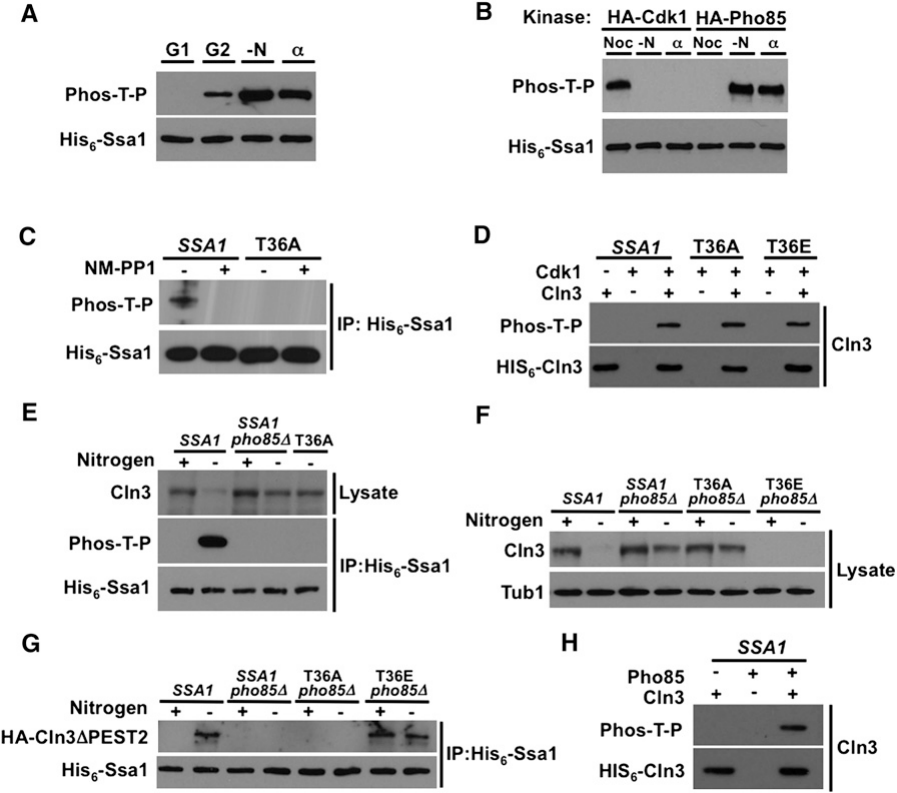
Ssa1 Is Phosphorylated by CDKs Cdk1 and Pho85 (A) Ssa1 is phosphorylated in G2 or in response to environmental conditions. Cells expressing HIS_6_-Ssa1 were either sorted into G1 or G2/M populations via flow cytometry or subjected to nitrogen starvation (-N) or αF stimulation (α). HIS_6_-Ssa1 was purified and T36 phosphorylation was assessed with anti-Phos-T-P antibody. (B) Cdk1 and Pho85 phosphorylate Ssa1 in vitro. Recombinant His_6_-Ssa1 was used as a substrate in an in vitro kinase assay with HA-Cdk1 or HA-Pho85 purified from cells treated with nocodazole (Noc), nitrogen starvation (-N) or αF (α). Ssa1 phosphorylation was assessed with anti-Phos-T-P antibody. (C) Cdk1 phosphorylates Ssa1 in vivo. Cdk-as1 cells expressing His_6_-Ssa1 (WT or T36A) were treated with 15 μg/ml nocodazole ± 10 μM 1-NM-PP1 for 3 hr. His_6_-Ssa1 was purified and T36 phosphorylation was assessed. (D) Phosphorylation of Cln3 by Cdk1 is independent of Ssa1 T36. Recombinant His_6_-Cln3 was used as a substrate in an in vitro kinase assay with HA-Cdk1 purified from *SSA1*, T36A or T36E cells treated with nocodazole. Cln3 phosphorylation was assessed with anti-Phos-T-P antibody. (E) Pho85 mediates Ssa1 T36 phosphorylation in nitrogen starvation. *SSA1* cells, *SSA1* cells bearing a *pho85*Δ mutation and T36A cells expressing His_6_-Ssa1 proteins were grown in media ± nitrogen source for 5 hr. Ssa1 was pulled down to determine T36 phosphorylation. (F) Ssa1 phosphorylation functions downstream of Pho85 with regard to Cln3 degradation. *SSA1* cells and *pho85*Δ mutant cells expressing *SSA1*, T36A or T36E were assayed for the effect of nitrogen starvation on Cln3 levels, revealing that Pho85 acts via phosphorylation of T36. (G) Pho85 regulates Ssa1-Cln3 interaction during nitrogen starvation. *SSA1* cells and *pho85*Δ mutant cells expressing His_6_-*SSA1*, T36A or T36E and stabilized, PEST mutant Cln3 were subjected to nitrogen starvation. Coprecipitation of HA-Cln3ΔPEST2 with His_6_-Ssa1 was assessed. (H) Cln3 is a direct Pho85 kinase substrate. Recombinantly expressed His_6_-Cln3 was used as a substrate in an in vitro kinase assay with HA-Pho85 purified from cells treated with nitrogen starvation. Cln3 phosphorylation was assessed with anti-Phos-S/T antibody. See also Figure S4.

**Figure S3:**
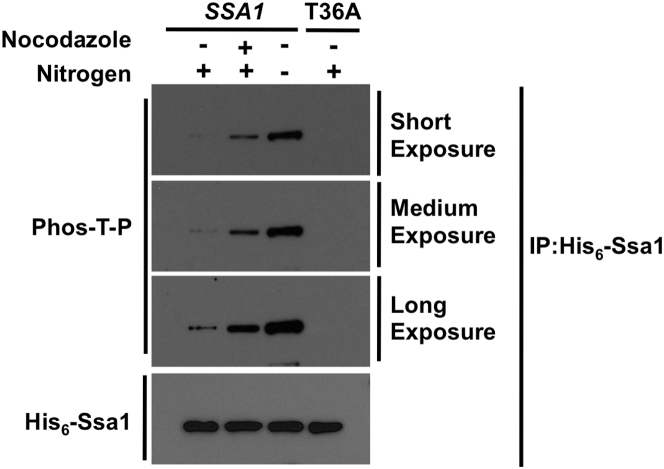
Ssa1 T36 Is Phosphorylated in Asynchronous Cells, Related to Figure 4 Cells expressing His_6_-tagged Ssa1 were treated with nocodazole or subjected to nitrogen starvation (5 hr). His_6_-tagged Ssa1 was purified and T36 phosphorylation was assessed with anti-Phos-T-P antibody.

Although T36 phosphorylation appeared absent from growing yeast cells compared to cells treated with αF (Figure 1H), overexposure of western blots detected potential low-level phosphorylation (Figure S3). To determine whether T36 phosphorylation might be cell cycle regulated, we isolated enriched G1 (unbudded) and G2/M (large budded) fractions from growing His_6_-*SSA1* cells via cytometric cell sorting. Ssa1 appeared unphosphorylated in G1 (high Cln), whereas T36 phosphorylation in G2/M (high Clb) was comparable to that after αF stimulation or nitrogen starvation (no Cln or Clb, Figure 4A). This raised the paradox that in a normal G1, when Cdk1-Cln1/2 is active, T36 remains unphosphorylated, but during G1 arrest in αF-treated and nitrogen-starved cells where Cdk1 is inactive, T36 is phosphorylated.

Because the CDKs Cdk1 and Pho85 share both specificity and substrates, we considered that both might be able to phosphorylate Ssa1 T36. We purified HA-Cdk1 or -Pho85 from cells treated with nocodazole (large budded mitotic checkpoint arrest, high Clb), nitrogen starvation, or αF and assessed their ability to phosphorylate recombinant Ssa1 in vitro. Only Cdk1 isolated from nocodazole-arrested cells and Pho85 purified from nitrogen-starved or αF-stimulated cells were able to phosphorylate Ssa1 (Figure 4B).

To confirm Cdk1-dependent phosphorylation of Ssa1 in vivo, we utilized a yeast strain expressing an analog-sensitive mutant Cdk1, Cdk1-as1 (Bishop et al., 2000). The inhibitor 1-NM-PP1 selectively binds to Cdk1-as1 and inhibits kinase activity within minutes. Cdk1-as1 cells expressing either His_6_-WT or His_6_-T36A were treated with nocodazole in the presence or absence of 1-NM-PP1. Inhibition of Cdk1-Clb kinase activity in vivo led to loss of T36 phosphorylation (Figure 4C). Cln3 is a direct substrate for Cdk1, marking it for destruction (Tyers et al., 1992). We asked whether Ssa1-Cln3 interaction may be required for Cdk1 to target Cln3 for phosphorylation. HA-Cdk1-Clb complexes were purified from *SSA1*, T36A and T36E cells treated with nocodazole and assessed for ability to phosphorylate recombinant His_6_-Cln3 in vitro. Suggesting that phosphorylation of Cln3 by Cdk1-Clb does not require Ssa1-Cln3 interaction, Cln3 phosphorylation was similar independent of whether any coprecipitated Ssa1 was WT, T36A, or T36E (Figure 4D).

**Figure S4:**
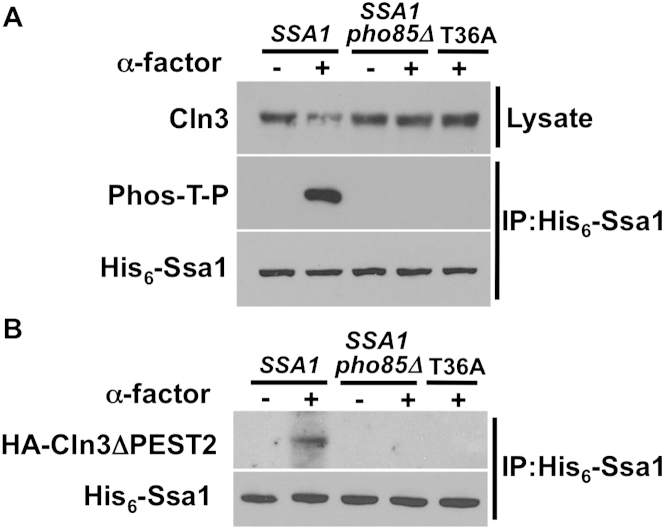
The CDK Pho85 Mediates Cln3 Degradation in Response to Pheromone, Related to Figure 4 (A) Pho85 mediates Ssa1 T36 phosphorylation in αF-treated cells. WT and *pho85*Δ mutant cells expressing His_6_-tagged Ssa1 were treated with αF. Total Cln3 levels were assessed. Ssa1 was purified and phosphorylation of Ssa1 determined. (B) Pho85 regulates Ssa1-Cln3 interaction in response to mating pheromone. WT and *pho85*Δ mutant cells expressing His_6_-tagged Ssa1 and stabilized, PEST mutant Cln3 were treated with αF and examined for Ssa1-Cln3 interaction.

To confirm the role of Pho85 in vivo, we examined Ssa1 phosphorylation and Cln3 accumulation in nitrogen-starved or αF-treated *pho85*Δ cells (Figure 4E; Figure S4A). Much like T36A cells under either condition, T36 phosphorylation was lost and Cln3 was stabilized in *SSA1* cells lacking Pho85. However, expression of T36E in *pho85*Δ triggered degradation of Cln3, placing Pho85 upstream of Ssa1 with regard to Cln3 regulation (Figure 4F). As expected, lack of Pho85 also abolished the binding of stabilized Cln3 to Ssa1 in nitrogen-starved or αF-treated *SSA1* cells (Figure 4G; Figure S4B).

Removal of the PEST domain stabilizes Cln3 even under conditions such as nitrogen starvation, where Cdk1 activity is low (Gallego et al., 1997), implying that a second kinase may phosphorylate Cln3 to promote degradation. Consistent with a dual role for Pho85 as an Ssa1 and Cln3 kinase, HA-tagged Pho85 purified from nitrogen-starved cells could directly phosphorylate recombinant His_6_-Cln3 (Figure 4H). Taken together, the results suggest that, (1) both Cdk1 and Pho85 can target Cln3 for destruction via direct phosphorylation of both Ssa1 and Cln3 and, (2) Pho85 activity prevents Cln3 accumulation during nitrogen starvation and αF stimulation.

### Roles of Ssa Proteins and Pcl Cyclins in Pho85-Dependent Regulation of Cln3

**Figure 5:**
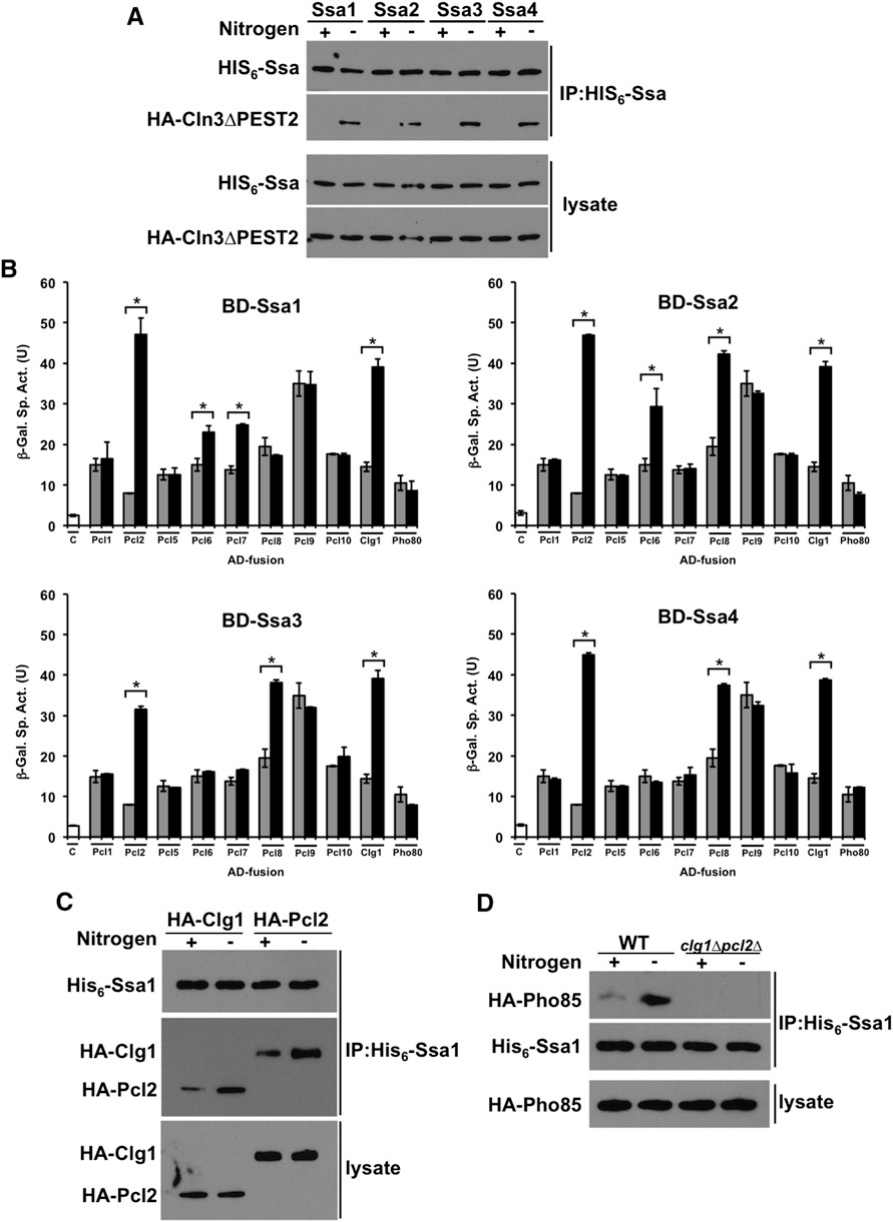
Ssa Proteins 1, 2, 3 and 4 Interact with Cln3 and a Specific Complement of Pho85 Cyclins (A) Ssa1-4 bind Cln3 upon nitrogen starvation. Cells expressing His_6_-Ssa1, 2, 3, and 4 and stabilized, PEST mutant Cln3 were grown in media ± nitrogen source for 5 hr. Coprecipitation of HA-Cln3ΔPEST2 with His_6_-Ssa1 was assessed. (B) Ssa1-4 interact with Pcl proteins in yeast two-hybrid assays. β-galactosidase activity was measured in protein extracts obtained from PJ694a/α cells transformed with the appropriate AD-Pcl and BD-Ssa1 fusions. Data in white bars represent AD-control fusion versus BD-Ssa. The data in grey represent cells transformed with BD-control versus AD-Pcl and data in black represent the BD-Ssa1 versus AD-Pcl interaction. Each value represents the mean ± SD (n = 3). Bars with ^∗^ represent significant Ssa-Pcl interactions as compared to control as indicated by brackets (p < 0.01). (C) Ssa1 interacts with Clg1 and Pcl2. Cells expressing His_6_-Ssa1 and either HA-Clg1 or HA-Pcl2 were grown in media ± nitrogen source for 5 hr. Ssa1 was pulled down and coprecipitation of Clg1 or Pcl2 with Ssa1 was determined. (D) Interaction of Pho85 with Ssa1 is dependent on Clg1 and Pcl2. WT and *clg1*Δ *pcl2*Δ mutant cells expressing His_6_-Ssa1 and HA-Pho85 were grown in media ± nitrogen source for 5 hr. Ssa1 was pulled down and coprecipitation of Pho85 was assessed. See also Figure S5.

T36 is conserved among the yeast Ssa proteins (Figure 1A). Thus, we tested whether, like Ssa1, Ssa2, 3, or 4 might serve as alternative Pho85 substrates and similarly associate with Cln3 upon nitrogen starvation. Indeed, isolation of His_6_-Ssa1, 2, 3, or 4 from cells also expressing stabilized Cln3 demonstrated increased Cln3 binding to each Ssa protein after nitrogen starvation (Figure 5A).

Like Cdk1 regulation by Clns and Clbs, Pho85 substrate specificity is determined through Pcl cyclin protein binding (Huang et al., 2007; Measday et al., 1997; Moffat et al., 2000). To detect which Pcl(s) might activate Pho85 to phosphorylate the Ssa proteins, we assessed physical interaction between Pcl proteins and Ssa1, 2, 3, and 4 by yeast two-hybrid analysis. Ssa1 associated most strongly with Clg1 and Pcl2 and to a lesser extent Pcl6 and Pcl7 (Figure 5B). Much like Ssa1, Ssa2, 3, and 4 all interacted strongly with Clg1 and Pcl2 but displayed distinct patterns of binding to other Pcl cyclins. Ssa2 bound to Pcl6 and 8, whereas Ssa3 and 4 only bound to Pcl8, suggesting specificity with respect to interaction with Pho85-cyclin complexes.

**Figure S5:**
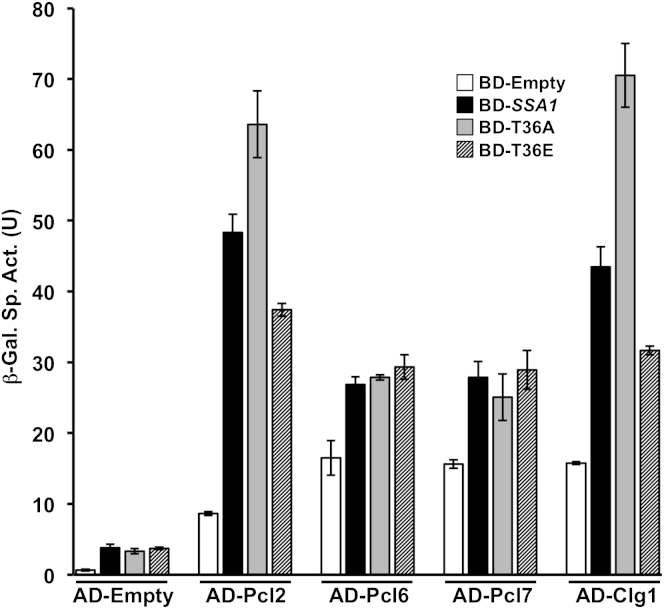
T36 Phosphorylation Alters Interaction between Ssa1 and a Subset of Pcl Proteins in Yeast Two-Hybrid Assays, Related to Figure 5 β-galactosidase activity was measured in protein extracts obtained from PJ694a/α cells transformed with the appropriate AD-Pcl and BD-Ssa1 fusions. Data shown are the average and SD (n = 3).

Suggesting that Clg1 and/or Pcl2 may target Pho85 to Ssa proteins as a substrate, His_6_-Ssa1 isolated from cells expressing HA-Clg1 or HA-Pcl2 displayed enhanced binding of Ssa1 to Clg1 and Pcl2 upon low nitrogen stimulation (Figure 5C). In turn, binding of Pho85 to Ssa1 induced by nitrogen starvation was lost in cells lacking both the Clg1 and Pcl2 cyclins (Figure 5D). Many kinases lose affinity for substrates after their phosphorylation. Further, consistent with a role in T36 phosphorylation, Clg1 and Pcl2 interaction in the two-hybrid assay was strengthened against T36A but decreased by T36E (Figure S5). Suggesting roles unrelated to Ssa1 phosphorylation, two-hybrid interactions of Pcl6 and 7 were independent of T36 mutation (Figure S5). These data suggest that Clg1 and/or Pcl2 may serve as the Pcl proteins that activate Pho85 to bind and phosphorylate Ssa proteins and target Cln3 for degradation.

### Phosphorylation of Hsc70 Alters Cyclin D1 Binding, Stability, and Cyclin D1-CDK-Mediated Signaling

**Figure 6:**
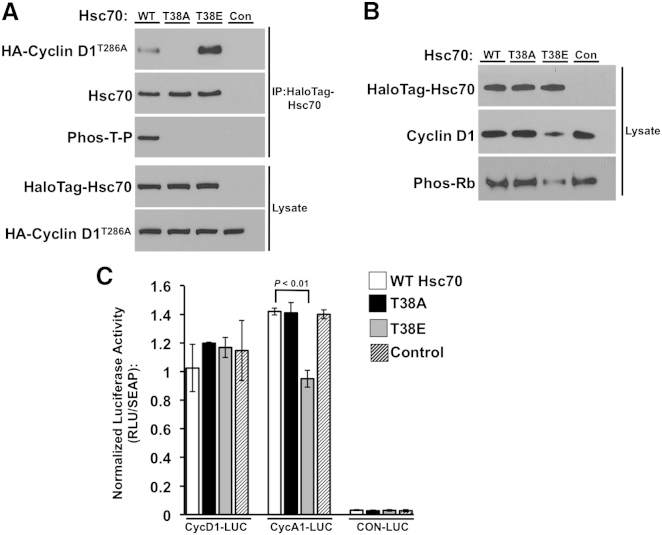
Phospho-Regulated Chaperone-Mediated Cyclin Destruction Is Conserved in Mammalian Cells (A) Mammalian Hsc70 is phosphorylated on T38, altering Cyclin D1 binding. Hsc70 was pulled down from HEK293 cells previously transfected with HaloTag-Hsc70 WT, T38A, T38E or empty control vector along with stabilized HA-Cyclin D1^T286A^. Hsc70 phosphorylation and interaction with Cyclin D1 were determined by western blot. (B) Overexpression of T38E Hsc70 alters Cyclin D1 accumulation and Rb protein phosphorylation. HEK293 cells were transfected with HaloTag-Hsc70 WT, T38A, T38E or empty control vector. After 72 hr, cells were harvested and levels of Cyclin D1 and Ser780 phosphorylated Rb were determined by western blot. (C) Overexpression of T38E Hsc70 alters Rb-mediated transcription. HEK293 cells were cotransfected in triplicate with HaloTag-Hsc70 WT, T38A, T38E or empty control vector and promoter-luciferase constructs to report expression of Cyclin D1 or Cyclin A1 or a negative control. After 72 hr, luciferase secreted into the media was assayed. Normalized luciferase activity is calculated by dividing luciferase-based luminescence by constitutively expressed alkaline phosphatase produced from the same construct. Each value represents the mean ± SD (n = 3). See also Figure S6.

**Figure S6:**
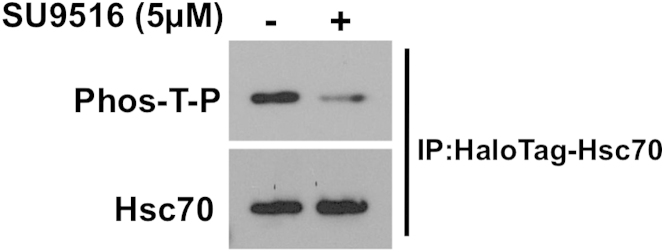
Chemical Inhibition of CDKs Results in Decreased Hsc70 T38 Phosphorylation, Related to Figure 6 HEK293T cells transfected with HaloTag-Hsc70 were grown in media lacking or supplemented with 5μM SU9516 for 24 hr. HaloTag-Hsc70 was purified and Hsc70 T38 phosphorylation was assessed with anti-Phos-T-P antibody.

Overall, Hsp70 family members are highly conserved. Among them, Ssa1 in yeast and Hsc70 in mammalian cells are considered to be functional homologs (Jaiswal et al., 2011). The Ssa1 T36 consensus CDK site is conserved in Hsc70 as T38 (Figure 1A) and Cyclin D1, the mammalian homolog of Cln3, has been shown to bind to Hsc70 (Diehl et al., 2003), raising the question of whether chaperone-mediated cyclin destruction may be conserved from yeast to human. Purification of HaloTag-Hsc70 WT, T38A, and T38E proteins overexpressed in HEK293T cells and probed with the anti-Phos-T-P CDK phosphosite antibody revealed Hsc70 phosphorylation at T38 (Figure 6A). Consistent with CDK-mediated phosphorylation, incubation with the CDK inhibitor SU9516 moderately decreased T38 phosphorylation (Figure S6).

To examine whether T38 phosphorylation may regulate the Hsc70-Cyclin D1 interaction, we utilized a stabilized Cyclin D1 mutant, T286A (Newman et al., 2004). Cells cotransfected with HA-tagged Cyclin D1^T286A^ and HaloTag-Hsc70 WT, T38A, or T38E were assessed for Hsc70-Cyclin D1 association via HaloTag pulldown. Consistent with regulation of Ssa1-Cln3 interactions by T36 phosphorylation in yeast, we observed decreased Cyclin D1^T286A^ binding to T38A but increased interaction with T38E, relative to WT (Figure 6A).

To test whether Hsc70 binding promotes Cyclin D1 degradation, we transfected cells to overexpress HaloTag-Hsc70 WT, T38A, or T38E or empty vector and examined for Cyclin D1 abundance after 72 hr. Although no change was observed in cells transfected with vector, WT, or T38A, Cyclin D1 levels were appreciably lower in cells expressing T38E (Figure 6B). CDK4/6-Cyclin D1 complexes phosphorylate the Rb tumor suppressor, a functional homolog of Whi5, on multiple sites, including S780. Using antiphospho-S780 antibody, we measured Rb phosphorylation in lysates from cells expressing HaloTag-Hsc70 WT, T38A, or T38E. Consistent with their decreased Cyclin D1 levels, cells expressing T38E displayed loss of Rb phosphorylation (Figure 6B). Rb phosphorylation permits transcriptional induction of multiple genes including Cyclin A1. As predicted from the lower Cyclin D1 and phospho-Rb levels, promoter-luciferase assays revealed decreased Cyclin A1 expression only in cells overexpressing Hsc70 T38E (Figure 6C).

These data suggest functional conservation between Ssa proteins and Hsc70, in which CDK-dependent phosphorylation of the chaperones promotes G1 cyclin binding and degradation to mediate G1/S cell cycle regulation.

## Discussion

### Regulation of Hsp70 through Phosphorylation

The prevailing view of Hsp70 is that regulation is achieved via a combination of altered expression of Hsp70, subtle differences among Hsp70 isoforms and the diversity of cochaperones that recruit specific clients. Here, we describe a mode of Hsp70 chaperone regulation mediated by direct phosphorylation, resulting in a dramatic switch in Hsp70-client interactions. Via proteomic analysis of the Ssa1 interactome, we observed a distinct pattern in the association of cochaperones and clients upon Ssa1 T36 phosphorylation. Among cochaperones, phosphorylation of T36 similarly decreased association of the J-protein Ydj1 and the Hsp70 nucleotide exchange factors Sse1 and 2. This is notable, given the complementary roles of J-proteins and Hsp70 NEFs in accelerating Hsp70 ATPase activity (Kampinga and Craig, 2010).

**Figure 7:**
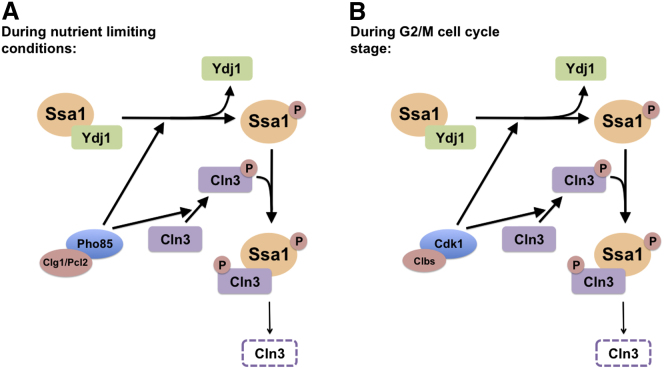
Model for Regulation of the Cell Cycle through Ssa1 Phosphorylation (A) In response to nitrogen starvation or exposure to prolonged αF mating pheromone, Ssa1 is phosphorylated on T36 by Pho85 activated by Pcl cyclins Clg1 and Pcl2. T36 phosphorylation triggers Ydj1 dissociation and promotes binding of Cln3, which may be phosphorylated by Pho85 on the PEST domains. Binding to Ssa1 and phosphorylation promote degradation of Cln3, decreasing G1/S cell cycle transcription and prolonging G1. (B) In G2/M phase, Cdk1 activated by Clb cyclins phosphorylates Ssa1, driving exchange of Cln3 for Ydj1, and targets Cln3 PEST domains. Both processes drive Cln3 degradation, preventing accumulation of Cln3 and resetting the cell for the next G1.

We do not infer that phosphorylation of T36 simply inactivates Hsp70, insofar as the effect is to decrease binding of the J-protein cochaperone Ydj1 while leaving interaction with a second J-protein, Sis1, relatively intact. In turn, our data suggest that the reciprocal binding of Ydj1 and the G1 cyclin Cln3 to Ssa1 described by Vergés et al. (2007) is mediated by T36 phosphorylation. Exchange of Ydj1 for Cln3 on Ssa proteins has been suggested as the key step that allows Cln3 to leave the ER, transit to the nucleus, and promote G1/S progression, but the switch-like kinetics of the START event do not favor a simple competition model. Our data offer a candidate mechanism underlying the observed Cln3/Ydj1 exchange, but suggest added complexity (Figure 7). Preventing the displacement of Ydj1 from Ssa proteins via the T36A mutation allows Cln3 protein to accumulate, much like the phenotype of mutation of the inhibitory J domain of Cln3 as reported by Vergés et al. (2007). In turn, the T36E mutant phenotype indicates that the Cln3 association with phosphorylated Ssa proteins mediates Cln3 destruction.

Our new data may help resolve the apparently conflicting views of Ydj1 function in prior literature. Although loss of Ydj1 results in decreased Cdk1-mediated phosphorylation of the Cln3 PEST domain and stabilization of Cln3 (Yaglom et al., 1996), *ydj1*Δ cells are shifted toward G1, a defect suppressed through overexpression of Cln3 (Vergés et al., 2007). The decreased Cln3 phosphorylation and lower Ssa1 T36 phosphorylation in cells lacking Ydj1 may each be explained by a decrease in Cdk1 (and perhaps Pho85) abundance in *ydj1*Δ cells (Mandal et al., 2008). Nonetheless, in cells lacking Ydj1, the Ssa1-Cln3 interaction becomes constitutive, perhaps limiting Cln3 access to the nucleus, and thereby partially uncoupling PEST domain phosphorylation, Cln3 stability, and Cdk1-Cln3 activity.

Our data suggest that the phosphorylation of the Cln3 PEST domain required for its degradation may be independent of Ssa1 binding. Bacterially expressed Cln3 can still be phosphorylated in vitro by Cdk1 purified from T36A cells. Additionally, Cln3 remains stabilized in cells where Cln3-Ssa1 interaction is made constitutive but phosphorylation of Cln3 cannot occur, such as T36E cells expressing Cln3 lacking PEST domains. Irrespective of dependency, we find that phosphorylation of Cln3 and the Cln3-Ssa1 interaction are each necessary but neither individually sufficient for degradation of Cln3.

A significant constraint on models is that the low abundance of Cln3 relative to Ssa1 or Ydj1 argues against a simple competition mechanism. We imagine a large pool of Ssa proteins, which may shift dynamically between dephosphorylated and phosphorylated states, depending on cell signals and/or cell cycle progression.

In turn, whereas the T36E mutation phenocopied a deletion of *CLN3* in several respects, it is striking that the T36A mutation displayed such a subtle phenotype under normal growth conditions. Were CDK-dependent Ssa phosphorylation the sole determinant of Cln3 abundance, we might anticipate that the T36A mutant would phenocopy *CLN3-1*, a hyperstabilized truncation mutant (Tyers et al., 1992). Unlike *CLN3-1*, Cln3 protein does not accumulate significantly in T36A under normal growth conditions, but fails to be destabilized under conditions that normally trigger Cln3 degradation. We infer that the nearly normal Cln3 levels in T36A may reflect alternative pathways for Cln3 degradation and/or the action of other mechanisms regulating Cln3 expression and abundance.

### Direct Control of the Cell Cycle by CDKs through Chaperone Phosphorylation

The CDKs Pho85 and Cdk1 are known to have overlapping substrate specificities and can phosphorylate the same residues in their targets (Huang et al., 2007; Kung et al., 2005; Mazanka and Weiss, 2010). In the case of Ssa chaperones, we have uncovered a mechanism where a single CDK consensus site on Ssa1, T36, can be phosphorylated by each CDK in response to specific cellular conditions. Thereby, both Cdk1 and Pho85 can promote Cln3 degradation, though under distinct circumstances.

Cdk1 has long been thought to control Cln3 degradation by direct phosphorylation at multiple sites within the carboxyl-terminal PEST domain (Tyers et al., 1992). Our data offer a second, indirect role for CDKs in Cln3 stability, mediated by Ssa protein phosphorylation. Significantly, we are able to detect Cdk1-mediated Ssa1 phosphorylation in vivo only in G2/M and in vitro only with Cdk1 complexes isolated from nocodazole-arrested cells. One implication is that Cln3 stability and activity may be maintained despite accumulation of Cdk1-Cln1/2 complexes by their inability to phosphorylate Ssa proteins. As such, that T36 is a substrate of Cdk1-Clb cyclin complexes, leading to Cln3 degradation, offers a novel mechanism for differentiating gene expression between G1 and S/G2/M in the yeast cell cycle. In turn, Ssa phosphorylation initiated by Cdk1-Clb complexes may persist through anaphase, so that daughter cells are born in G1 with destabilized Cln3.

In searching for a CDK that phosphorylates Ssa proteins in G1, we uncovered a role for the stress-activated kinase Pho85 in inhibiting G1/S cell cycle progression. We found that Pho85 can phosphorylate Ssa1 in response to nitrogen starvation or pheromone stimulation, leading to Cln3 degradation and a delay in S phase onset (Figure 7). We also uncovered an unanticipated role for Pho85 in the direct phosphorylation of Cln3. This may explain how PEST-mediated destruction of Cln3 can still occur in conditions where Cdk1 activity is low, such as nitrogen starvation. This is particularly interesting in light of previously established roles for Pho85 in promoting G1/S progression through direct phosphorylation of Whi5 (Huang et al., 2009) and inhibition of Sic1 (Wysocki et al., 2006). Opposing roles for Pho85 kinase have previously been ascribed to association with distinct Pcl cyclin partners. Clg1 and Pcl2 interact with Ssa proteins and appear to be candidates for the Pcls that direct Pho85 to target Ssa proteins as substrates and delay G1/S progression. Notably, Pcl2 expression is uniquely induced by pheromone stimulation (Measday et al., 1997). By contrast, neither Pcl9, a cyclin that binds Pho85 to promote Whi5 phosphorylation (Tennyson et al., 1998), nor Pho80, a candidate for the cyclin mediating Sic1 phosphorylation (Wysocki et al., 2006), appeared to interact with Ssa proteins, consistent with Pcl expression determining whether Pho85 serves as a positive or negative regulator of cell cycle progression. The Ssa1-Pcl6/7 interaction observed is also interesting, particularly as our proteomic analysis revealed a dependence of the interaction of Glc7, a regulator of glycogen accumulation with Ssa1 on T36 phosphorylation. Notably, Pcl6/7 activate Pho85 to regulate glycogen metabolism, via Glc7 (Huang et al., 1998; Tan et al., 2003; Timblin et al., 1996), suggesting further connections between Ssa protein phosphorylation, metabolism, and cell-cycle progression.

The Ssa1 phosphorylation site T36 is present in each Ssa isoform, suggesting that Ssa1 through 4 may be similarly regulated by CDK phosphorylation. In our studies, although some Pcl cyclins appeared to bind all four isoforms, isoform-specific interactions were also observed between Ssa proteins and Pcls, suggesting both overlapping and distinct functions for isoforms in cell-cycle control. A pattern of functional redundancy and specificity among Ssa proteins has previously been observed for both yeast prion propagation and protein folding activities (Sharma et al., 2009; Sharma and Masison, 2011).

### Phosphorylation of Hsp70 Family Members as a Conserved Determinant of G1 Cyclin Function

The mammalian homolog of Cln3, Cyclin D1, is an important regulator of the G1/S progression in normal cells and commonly deregulated in cancer (Alao, 2007; Enserink and Kolodner, 2010). Hsc70 associates with Cyclin D1 and is present as an element of the active CDK4-Cyclin D1 complex, although a specific functional role in the complex remains to be described (Diehl et al., 2003; Trotter et al., 2001). We found that CDK-dependent phosphorylation of Hsc70 promotes Cyclin D1 binding and destruction, thereby lowering Rb phosphorylation and Cyclin A expression. Promoting Hsc70 T38 phosphorylation may provide a novel target for cancer therapeutics, via destabilizing Cyclin D to slow tumor cell proliferation.

Overall, this study reveals an unanticipated layer of Hsp70 chaperone regulation mediated by phosphorylation. Much like recent work with Hsp90 (Mollapour et al., 2010), we find that client binding can be regulated through posttranslational modification, although here with critical consequences to cell-cycle progression. We show that Hsp70 chaperones serve as more than simple housekeeping proteins but are dynamically activated in response to cell-cycle progression and environmental conditions and serve a critical role as effectors that transduce cell signaling to cell cycle control.

## Experimental Procedures

### Yeast Culture, Strains, and Plasmids

A list of yeast strains and plasmids used in this study is available in Tables S2 and S3. Standard methods were used for yeast, bacteria, and mammalian cell culture, as described in Extended Experimental Procedures. For αF stimulation or nitrogen starvation experiments, cells were treated as described (Gallego et al., 1997).

### Analysis of the Ssa1 Interactome by Mass Spectrometry

Yeast cells expressing either His_6_-Ssa1 WT or T36A as the sole Ssa1 protein were grown to mid-log phase, treated with 20 μM αF for 2.5 hr and lysed. His_6_-Ssa1 WT or T36A interactomes were purified on His-Tag Isolation Dynabeads (Invitrogen), separated by SDS-PAGE and subjected to proteomic analysis. Briefly, gel slices were subjected to in-gel proteolysis; tryptic peptides were purified, differentially labeled by carboxyl-terminal ^18^O exchange, and analyzed via LC-MS/MS as described in Extended Experimental Procedures.

### Flow Cytometry

Analysis of DNA content, cell size measurement, and cell sorting were performed as described (Jorgensen et al., 2002).

### Protein Analysis, Pull-Down, and Immunoblotting

Extracts of total protein were prepared as described (Kamada et al., 1995). Affinity-based enrichment by pull-down and detection by western blotting are detailed in Extended Experimental Procedures.

### β-Galactosidase Assays

Cells transformed with the appropriate promoter-LacZ fusions were grown to mid-log phase and total protein was extracted. 100 μg of cell lysate were used in the assay as described (Kamada et al., 1995).

Extended Experimental Procedures **Yeast Culture, Strains, and Plasmids**

The *S. cerevisiae* strains used in this study are listed in Table S2. Yeast cultures were grown in YEPD (1% yeast extract, 2% peptone, 2% dextrose) or SD (0.67% yeast nitrogen base, 2% dextrose) supplemented with the appropriate nutrients to select for plasmids and gene replacements.

For serial dilutions, cultures were diluted to an optical density at 600 nm of 1.0 and 5 μl aliquots of a 10-fold dilution series were spotted onto appropriate media. Growth was monitored over 3 to 5 days at 25°C.

*CLN3*, *WHI5*, *Pho85*, *BCK2 and YDJ1* were deleted in the SKY3057 *ssa1-4*Δ background using the *HpHMX4* cassette PCR amplified from pAG32 as described (Goldstein and McCusker, 1999). Primer sequences are available on request. Genotypes were confirmed by PCR analysis.

*E. coli* DH5α was used to propagate all plasmids. *E. coli* cells were cultured in Luria Broth (1% tryptone, 0.5% yeast extract, 1% NaCl) and transformed to carbenicillin resistance by standard methods. Plasmids are listed in Table S3.

Plasmids expressing Ssa1 phospho-site mutants were created using the Quickchange site-directed mutagenesis kit (Agilent Technologies). Primer sequences are available on request. Mutations were confirmed by DNA sequencing.

**Yeast Halo Assay**

Yeast cells were grown overnight to stationary phase. The following day the culture was diluted 1:1000 and 150 μl of cells were spread onto a YPD plate. After the plate had been incubated for 2 hr at 30°C, 10 μl of 5 μg/ml of synthetic α factor peptide (WHWLQLKPGQPNleY) in DMSO was spotted onto circular filter paper and placed onto the aforementioned media. The plate was incubated for 2 days at 30°C and then photographed.

**Yeast Two Hybrid Analysis**

PJ694a cells (Uetz et al., 2000) were transformed with the appropriate Two-hybrid plasmids (see Table S2). Interaction strength was measured via β-galactosidase assays (Millson et al., 2005).

**Purification of HIS_6_-Tagged Ssa1 from Yeast**

HIS_6_-tagged Ssa1 expressed in strain SKY3057 as the sole Ssa protein was isolated as follows. Protein was extracted via bead beating in 500 μl Binding/Wash Buffer (50 mM Na-phosphate pH 8.0, 300 mM NaCl, 0.01% Tween-20). 200 μg of protein extract was incubated with 50 μl His-Tag Dynabeads (Invitrogen) at 4°C for 15 min. Dynabeads were collected by magnet then washed 5 times with 500 μl Binding/Wash buffer. After final wash, buffer was aspirated and beads were incubated with 100 μl Elution buffer (300 mM Imidazole, 50 mM Na-phosphate pH 8.0, 300 mM NaCl, 0.01% Tween-20) for 20 min, then beads were collected via magnet. The supernatant containing purified HIS_6_-Ssa1 was transferred to a fresh tube, 25 μl of 5x SDS-PAGE sample buffer was added and the sample was denatured by boiling for 5 min at 95°C. 10 μl of sample was analyzed by SDS-PAGE. 10 μg of whole-cell extract was used as loading control.

**Western Blotting**

Protein extracts were made as described (Kamada et al., 1995). 20 μg of protein was separated by 4%–12% SDS-PAGE (Invitrogen). Cln3 was detected by immunoblot analysis with polyclonal goat anti-Cln3 antibodies (yN-19, Santa Cruz Biotechnologies) at a 1:2,000 dilution. Chicken anti-goat IgG-HRP conjugate secondary antibody (Millpore) was used at a 1:5000 dilution. HIS_6_-tagged proteins were detected using Tetra-HIS antibody (QIAGEN) at a dilution of 1:3000 and secondary antibodies were used at 1:6,000. Ssa1 Thr 36 phosphorylation was detected using Phospho-Threonine-Proline Mouse mAb (Cell Signaling Technologies) at a 1:500 dilution and anti-mouse IgG-HRP conjugate (GE Healthcare) at a 1:4,000 dilution. Antiserum to Sis1 was a kind gift from Elizabeth Craig (UW Madison). Samples were separated by SDS-PAGE and immunoblotted using the conditions described in (Aron et al., 2005). HALO-Hsc70 was detected using Anti-HaloTag pAb (G9281, Promega) at a 1:3000 dilution and anti-rabbit IgG-HRP conjugate (GE Healthcare) at a 1:5000 dilution. Phosphorylated Rb was detected using Phospho-Rb (Ser780) Antibody (9307, Cell Signaling) at a 1:500 dilution and anti-rabbit IgG-HRP conjugate (GE Healthcare) at a 1:4,000 dilution. Wherever possible, blots were stripped and re-probed with the relevant antibodies using Restore Western Blot Stripping Buffer (Thermo). Otherwise, samples from same experiment were run on parallel gels and the blots were processed identically.

**Expression and Purification of Recombinant HIS_6_-Tagged Cln3 from Bacteria**

BL21 cells transformed with plasmid pRSETA *CLN3* were grown to an OD_600_ of 0.6. His_6_-tagged Cln3 expression was induced by addition of 1 mM IPTG and incubation of cells at 30°C for 4 hr. Cells were lysed in P-PER reagent (with protease inhibitors) and the lysate was clarified by centrifugation, HIS_6_-tagged Cln3 was purified using His-Tag Dynabeads (Invitrogen). Eluted protein was dialyzed via a 20,000 MWCO Slide-A-Lyzer cassette (Thermo) to remove imidazole.

**In Vitro Kinase Assays**

For kinase assays, 100 μg yeast lysates were incubated with anti-HA antibody conjugated beads (Sigma) for 2 hr at 4°C. Beads were washed 4 times with fresh lysis buffer, then one final time in kinase assay buffer (100 mM Tris-HCl [pH 8.0], 100 mM NaCl, 10 mM MgCl_2_, 20% glycerol). The supernatant was removed and the beads were supplemented with 20 μl of kinase assay buffer along with 10 μg of recombinant Cln3 (see previous) or Ssa1 substrate, expressed and purified from *E. coli* as described (Rowlands et al., 2010). After 3 min, 100 μM ATP was added to initiate the kinase reaction. The reaction was allowed to proceed for 30 min at 30°C and terminated by addition of 50 μl SDS Laemmli buffer followed by boiling for 5 min. Samples were separated on a 4%–12% SDS-PAGE gel and immunoblotted using the Phospho-Thr-Pro antisera as described above. Blots were stripped using Restore Western Blot Stripping Buffer (Thermo) and re-probed with Tetra-HIS antibody.

**Ssa1 ATPase Assays**

*E. coli* BL21 (DE3) codon+ cells transformed with plasmids for Ssa1 (WT, T36A and T36E) were grown in 2YT medium at 37°C until OD600 = 0.6–0.8. Protein expression was induced with 0.2 mM IPTG and grown overnight at 16°C. Cells were harvested and lysed in buffer A (20 mM HEPES buffer [pH 7.5], containing 300 mM KCl, 20 mM MgCl_2_, 5% Glycerol, 2 mM β-mercaptoethanol and complete protease inhibitor cocktail (Roche)) using a JN-3000 plus French press (JNBIO). Cell debris was cleared by centrifugation (30,000 g) and the supernatant loaded on Chelating Sepharose FF metal affinity resin (GE). After washing with buffer A containing 100 mM imidazole, protein was eluted with buffer A containing 350 mM imidazole and dialyzed into buffer B (50 mM Tris-HCl buffer, pH7.5 containing 100 mM KCl, MgCl_2_ and 1 mM DTT). The protein was concentrated with a Millipore ultrafilter and loaded onto a 24 ml or 120 ml Superdex 200 column (GE) equilibrated with buffer B. The second peak of the elution was collected for ATPase assay. Protein purity was more than 99% as determined by SDS/PAGE and Coomassie staining. Protein concentration was measured by absorbance at 280 nm using the calculated extinction coefficient of 18,610 M-1 cm-1 for Ssa1 and its mutants. ATPase activity was measured using a coupled enzymatic assay, where the consumption of NADH was monitored by the absorbance at 340 nm. ATPase activity of Ssa1 was assayed in buffer B with 2 U ml-1 PK, 10 U ml-1 LDH and 4 mM PEP, an Ssa1 concentration of 1 μM and 500 μM ATP. The assays were carried out in thermostatted 1 ml cuvettes at 30°C using a Shimadzu UV-2501 spectrophotometer. After addition of 0.12 mM NADH oxidation was measured by monitoring the absorbance at 340 nm for 100 s before ATP was added and for another 500 s after ATP was added. The difference in slope between the 100 s initial baseline and the 500 s measurement represents the rate of ATP hydrolysis. The actual rate of ATP hydrolysis catalyzed by Ssa1 or its mutants was obtained by subtracting the rate of ATP self-hydrolysis (i.e., measured in the absence of Ssa1 or its mutants). *V* = (d*A340 nm*/d*t*)/ε340 nm, where the molar absorbance coefficient for NADH ε340 nm = 6200 M-1 cm-1.

**Isothermal Titration Calorimetry**

The heat of interaction was measured on an ITC_200_ microcalorimeter (Microcal), with a cell volume of 200 μL, under the same buffer conditions (20 mM Tris, pH 7.5, containing 6 mM MgCl_2_ and 5 mM NaCl) at 30°C. 10 3.8 μl aliquots of WT or mutant Ssa1 protein at 30 μM were injected into 350 μM of AMP-PNP. Heats of dilution were determined in a separate experiment by diluting protein into buffer, and the corrected data were fitted using a nonlinear least-squares curve-fitting algorithm (Microcal Origin) with three floating variables: stoichiometry, binding constant and change in enthalpy of interaction.

**Trypsin Digestion of Samples from SDS-PAGE Gels**

Gel lanes to be analyzed were excised from SDS-PAGE gels by razor blade and divided into ∼2.5 cm slices and chopped into ∼1 mm^3^ pieces. Each section was washed in water and destained using 100 mM ammonium bicarbonate pH 7.5 in 50% acetonitrile. A reduction step was performed by addition of 100 μl 50 mM ammonium bicarbonate pH 7.5 and 10 μl of 10 mM Tris(2-carboxyethyl)phosphine HCl at 37°C for 30 min. The proteins were alkylated by adding 100 μl 50 mM iodoacetamide and allowed to react in the dark at 20°C for 30 min. Gel sections were washed in water, then acetonitrile, and dried in a SpeedVac. Trypsin digestion was carried out overnight at 37°C with 1:50 enzyme-protein ratio of sequencing grade-modified trypsin (Promega) in 50 mM ammonium bicarbonate pH 7.5, and 20 mM CaCl_2_. Peptides were extracted with 5% formic acid and vacuum dried.

**Isotopic Labeling**

Thirty microliters of Tris-HCl Buffer Solution (10 mM of Tris-HCl, 150 mM NaCl, 20 mM CaCl_2_, pH 7.6) was added along with 10 μl of trypsin solution (2 ug of trypsin (Promega) in 100 μl of 50 mM ammonium bicarbonate, pH 7.5). The sample was dried, then 30 μl of 97% H_2_^18^O water (Cambridge Isotope Labs) was added to the dry tube. The sample tube was vortexed for 30 s and incubated overnight at 37°C. The reaction was stopped by adding 1 μl of 10% TFA solution. The sample was dried and stored at −80°C until analysis.

**HPLC for Mass Spectrometry**

The peptide samples were loaded to a 0.25 μl C_8_ OptiPak trapping cartridge custom-packed with Michrom Magic C8 (Optimize Technologies), washed, then switched in-line with a 20 cm by 75 μm C_18_ packed spray tip nano column packed with Michrom Magic C18AQ, for a 2-step gradient. Mobile phase A was water/acetonitrile/formic acid (98/2/0.2) and mobile phase B was acetonitrile/isopropanol/water/formic acid (80/10/10/0.2). Using a flowrate of 350 nl/min, a 90 min, 2-step LC gradient was run from 5% B to 50% B in 60 min, followed by 50%–95% B over the next 10 min, hold 10 min at 95% B, back to starting conditions and re-equilibrated.

**LC-MS/MS Analysis**

The samples were analyzed via electrospray tandem mass spectrometry (LC-MS/MS) on a Thermo LTQ Orbitrap XL, using a 60,000 RP survey scan, m/z 375-1950, with lockmasses, followed by 5 LTQ CAD scans on doubly and triply charged-only precursors between 375 Da and 1500 Da. Ions selected for MS/MS were placed on an exclusion list for 60 s. ReadW was used to convert .RAW files to .mzxml, which were imported into Comparative Proteomics Analysis System (CPAS). Peptides were assigned through X! Tandem database searches using the Swissprot database, under conditions of: Digestion enzyme = trypsin; 2.5 Da fragment ion mass tolerance; 1 Da parent ion tolerance; allowed modifications were oxidation of methionine, cyclization of glutamine and cysteine, single and double carboxyl terminal ^18^O and fixed cysteine carbamidomethyl. Results with less than 0.01 fractional delta mass were processed using Peptide Prophet, requiring a Peptide Prophet value of 0.9 or higher, and quantitation performed using XPRESS. Quantitated peptides were exported and combined into one Excel file. *Saccharomyces cerevisiae* proteins were compiled and average protein ratios (minimum 2 peptides per protein) were calculated using in-house software from the Hanash lab at Fred Hutchinson Cancer Research Center (Seattle). The data were normalized to SSA1 protein.

**Purification of HaloTag-Hsc70 from HEK293T Cells**

HEK293T cells were cultured in Dulbecco’s modified Eagle’s minimal essential medium (DMEM; Invitrogen, Carlsbad, CA, USA) supplemented with 10% fetal bovine serum (FBS; Invitrogen), 100 U/ml penicillin (Invitrogen) and 100 μg/ml streptomycin (Invitrogen). All cell lines were incubated at 37°C in a 5% CO_2_ containing atmosphere. Cells were transfected with a plasmid expressing HaloTag-Hsc70 and/or HA-Cyclin D1 (T286A) using the Lipofectamine 2000 transfection kit (Invitrogen). After 24 hr, protein was extracted and HaloTag-Hsc70 was purified using the HaloTag Mammalian Pull-Down System (Promega).

**Overexpression of HaloTag-Hsc70 in HEK293T Cells**

HEK293T cells were transfected with the appropriate expression constructs and grown for 72 hr. Cells were harvested and lyzed for western blotting.

**Measurement of Promoter-Luciferase Expression**

HEK293T cells were transfected with the appropriate luciferase reporter constructs (GeneCopoeia) using Lipofectamine 2000 transfection kit (Invitrogen). After 72 hr, 1ml of media was removed for analysis. Luciferase assays were performed as per manufacturer’s recommendations and both relative promoter-Luciferase and constitutively expressed Secreted Alkaline Phosphatase (SEAP) were measured. Normalized Luciferease activity was calculated by dividing the relative luciferase activity by SEAP.

## Supplemental Information

Table S1. Mass Spectrometry Data of T36A/WT Ssa1 Interactomes, Related to Figure 2 Peptides from Ssa1-T36A interactors were isotopically labeled with 18O, mixed 1:1 with wild-type interactor peptides, and analyzed by quantitative LC-MS/MS. The data were normalized to Ssa1 protein. The table lists the identified proteins by Swissprot protein accession number, Swissprot protein name, SGD name, mean peptide ratio of Ssa1-T36A/Ssa1-WT, ln mean peptide ratio, number of peptides identified, standard deviation (of ln mean) and p-value.

Document S1. Table S2 and S3

Document S2. Article plus Supplemental Information
