# Supplementary material for: CDK-Dependent Hsp70 Phosphorylation Controls G1 Cyclin Abundance and Cell-Cycle Progression
Source: Cell. 2012 Dec 7;151(6):1308–18. doi: 10.1016/j.cell.2012.10.051 (PMC3778871; doi:10.1016/j.cell.2012.10.051)
Supplement: Document S1. Table S2 and S3 [file mmc2.pdf]

Table S2. Yeast Strains Used in This Study

Strain	Genotype	Reference/Source
SKY3056	*MAT***a** (W303) *CDK1::cdk1-as1-pRS306 URA3*	Bishop et al., 2000
SKY3057	*MAT***a** (MH272) *ssa1Δ::trp1 ssa2::HisG ssa3::HisG ssa4::HisG* (*ssa1-4*) [YCPlac33 *SSA1*]	Jaiswal et al., 2011
SKY3058	*MAT***a** (MH272) *ssa1-4 cln3Δ::HpHMX4* [YCPlac33 *SSA1*]	This study
SKY3059	*MAT***a** (MH272) *ssa1-4 whi5Δ::HpHMX4* [YCPlac33 *SSA1*]	This study
SKY3060	*MAT***a** (MH272) *ssa1-4 pho85Δ::HpHMX4* [YCPlac33 *SSA1*]	This study
SKY3061	*MAT***a** (MH272) *ssa1-4 bck2Δ::HpHMX4* [YCPlac33 *SSA1*]	This study
SKY3062	MAT**a** (PJ69-4a) *trp1-901 leu2-3,112 ura3-52 his3-200 gal4Δ gal80Δ LYS2::GAL1-HIS3 GAL2-ADE2 met2::GAL7-lacZ*	This study
SKY3063	MAT**a** (PJ69-4a) pOAD *PCL1*	Uetz et al., 2000
SKY3064	MAT**a** (PJ69-4a) pOAD *PCL2*	Uetz et al., 2000
SKY3065	MAT**a** (PJ69-4a) pOAD *PCL5*	Uetz et al., 2000
SKY3066	MAT**a** (PJ69-4a) pOAD *PCL6*	Uetz et al., 2000
SKY3067	MAT**a** (PJ69-4a) pOAD *PCL7*	Uetz et al., 2000
SKY3068	MAT**a** (PJ69-4a) pOAD *PCL8*	Uetz et al., 2000
SKY3069	MAT**a** (PJ69-4a) pOAD *PCL9*	Uetz et al., 2000
SKY3070	MAT**a** (PJ69-4a) pOAD *PCL10*	Uetz et al., 2000
SKY3071	MAT**a** (PJ69-4a) pOAD *CLG1*	Uetz et al., 2000
SKY3072	MAT**a** (PJ69-4a) pOAD *PHO80*	Uetz et al., 2000
SKY3073	*MAT***a** S288c (BY4741) *his3 leu2 ura3 lys2*	Research genetics
SKY3074	*MAT***a** S288c (BY4741) *clg1Δ::KanMX4 pcl2Δ::HpHMX4*	This Study
SKY3075	*MAT***a** (MH272) *ssa1-4 ydj1Δ::HpHMX4* [YCPlac33 *SSA1*]	This Study

Table S3. Plasmids Used in This Study

**Plasmid**	**Description**	**Reference/Source**
pRS313	pRS313 (*HIS3* centromeric plasmid)	Sikorski and Hieter, 1989
pRS315	pRS315 (*LEU2* centromeric plasmid)	Sikorski and Heiter, 1989
pRS316	pRS316 (*URA3* centromeric plasmid)	Sikorski and Heiter, 1989
SKB4262	pAG32	Goldstein and McCusker, 1999
SKB4522	pC210 *SSA1*	Schwimmer and Masison, 2002
SKB4523	pC210 *ssa1-T36A*	This study
SKB4524	pC210 *ssa1-T36E*	This study
SKB4546	pC210 HIS_6-*SSA1*	This study
SKB4547	pC210 HIS_6-*ssa1-T36A*	This study
SKB4548	pC210-HIS_6-*ssa1-T36E*	This study
SKB4552	pRSETA *SSA1*	This study
SKB4584	pRSETA *ssa1-T36A*	This study
SKB4585	*CLN3-CYC1-lacZ*	Polymenis and Schmidt, 1997
SKB4586	p*HA-CLN3ΔPEST2*	Verges et al., 2007
SKB4587	p*tetO*$_7$ *HA-PHO85*	Wanke et al., 2005
SKB4588	p*GAL1 ZZ-HA-*HIS_6*-CDK1*	Open Biosystems
SKB4589	pGBT9; two-hybrid vector with Gal4-DBD	Clontech
SKB4590	pOAD; two-hybrid vector with Gal4-AD	Uetz et al. 2000
SKB4591	p*BD-SSA1*	Wegele et al., 2003
SKB4592	p*BD-SSA2*	Wegele et al., 2003
SKB4593	p*BD-SSA3*	Wegele et al., 2003
SKB4594	p*BD-SSA4*	Wegele et al., 2003
SKB4595	p*CMV HaloTag-HSC70*	Promega
SKB4596	p*CMV HaloTag-HSC70-T38A*	This study
SKB4597	p*CMV HaloTag-HSC70-T38E*	This study
SKB4598	p*CMV HA-Cyclin D1-T286A*	Addgene plasmid 11182
SKB4599	*CLN2* (-600 TO -400)-*CYC1-lacZ*	Kim et al., 2008
SKB4600	*pGAL1 WHI5-FLAG*	Costanzo et al., 2004
SKB4601	pRSETA *CLN3*	This study
SKB4602	p*BD-SSA1 T36A*	This study
SKB4603	p*BD-SSA1 T36E*	This study
SKB4604	p*GAL1 HA-CLG1*	Yang et al., 2010
SKB4605	p*GAL1 HA-PCL2*	Yang et al., 2010

SKB4606	*CYCLIN A1*-Luciferase	GeneCopoeia plasmid HPRM12202-PG04)
SKB4607	*CYCLIN D1*-Luciferase	GeneCopoeia plasmid (HPRM25565-PG04)
SKB4608	*Negative control*-Luciferase	GeneCopoeia plasmid (NEG-PG04)
SKB4509	p*GAL1 ZZ-HA-*HIS_6*-SSA1*	Open Biosystems
SKB4510	p*GAL1 ZZ-HA-*HIS_6*-SSA2*	Open Biosystems
SKB4511	p*GAL1 ZZ-HA-*HIS_6*-SSA3*	Open Biosystems
SKB4512	p*GAL1 ZZ-HA-*HIS_6*-SSA4*	Open Biosystems
SKB4513	pRS315-*HA-CLN3∆PEST2*	This study
